# Proposition of a corrected measure of the Lawton instrumental activities of daily living score

**DOI:** 10.1186/s12877-020-01995-w

**Published:** 2021-01-12

**Authors:** Marine Dufournet, Claire Moutet, Sarah Achi, Floriane Delphin-Combe, Pierre Krolak-Salmon, Virginie Dauphinot, the MEMORA group, Pierre Krolak-Salmon, Virginie Dauphinot, Florian Delphin-Combe, Zaza Makaroff, Denis Federico, Marie-Hélène Coste, Isabelle Rouch, Jean-Michel Dorey, Alexis Lepetit, Keren Danaila, Julien Vernaudon, Anthony Bathsavanis, Alain Sarciron, Yves Guilhermet, Sylvain Gaujard, Pierre Grosmaître, Claire Moutet, Mathieu Verdurand, Antoine Garnier-Crussard

**Affiliations:** 1 Clinical and Research Memory Centre of Lyon of Lyon (CMRR Lyon), Lyon Institute For Elderly (Institut du vieillissement I-Vie), Hospices civils de Lyon, Lyon, France; 2 Research Clinic Centre Aging Brain Frailty (CRC - VCF), Lyon Institute For Elderly (Institut du vieillissement I-Vie), Hospices civils de Lyon, Lyon, France; 3 University Lyon 1, INSERM, U1028, UMR CNRS 5292, Research Centre of Neurosciences of Lyon, Lyon, France; 4 Hôpital des Charpennes, 27 avenue Gabriel Péri, 69 100 Villeurbanne, France

**Keywords:** Dependency, Neurocognitive disorders, Dementia, Cognition, Reproducibility of results

## Abstract

**Background:**

We aimed to propose a correction of the Lawton instrumental activity of daily living (IADL) score to take into account the possibility to have never done some activities, and measured its agreement and reliability with the usual IADL score.

**Methods:**

A cross-sectional study was conducted in outpatients attending French memory clinics between 2014 and 2017. Lawton IADL, cognitive performance, diagnosis, neuropsychiatric symptoms, and sociodemographics characteristics were collected. A corrected IADL was calculated and its agreement with the usual IADL was assessed.

**Results:**

The study included of 2391 patients (79.9 years old, 61.7% female). Based on the usual IADL, 36.9% of patients had never carried out at least one of the activities. This proportion reached 68.8% for men and 17.7% for women. Women had a mean IADL higher than men: 4.72 vs 3.49, this difference decreased when considering the corrected IADL: 4.82 vs 4.26 respectively. Based on Bland-Altman method, 93.5% of observations lied within the limits agreement. The ICC between the 2 scores was 0.98. The relationships between patients’ characteristics and the IADL scores were similar, regardless the usual or corrected version.

**Conclusions:**

This corrected IADL score had an excellent degree of agreement with the usual version based the ICC. This simple correction could benefit both for the clinical practice by providing a more accurate description of the real clinical state of the patients allowing to manage them more precisely, and for research involving the evaluation of the functional abilities of patients.

## Background

Alzheimer’s disease or related disorders are a main cause of functional decline, and the diagnosis of major neurocognitive stage is defined as a significant cognitive decline which interfere with independence in activities of daily living, as presented in the Diagnosis and Statistical Manual of mental disorders (DSM-V) criteria [1]. Functional decline in older adults leads to institutionalization and increased health-care costs, as well as shifting many daily responsibilities to caregivers and increasing their burden [2–5]. The accuracy of the functional abilities assessment is therefore one of the key features in the diagnosis of neurocognitive disorders and their change, as well as in research studies which aim to assess the effect of interventions to delay functional decline [6, 7]. Among the several scales available to measure the level of functional abilities, and in the current lack of consensus, the Lawton Instrumental Activities of Daily Living (IADL) scale has been widely adopted, and is usually part of the evaluation in clinical practice in the memory centers in France [8, 9]. The Lawton IADL scale was developed to assess more complex abilities of daily living necessary for living independently in the community, there are nevertheless a few information on its psychometric proprieties [8, 10]. Additionally, the original Lawton IADL scale displays some limits since it does not take into account the possibility to have never done one or several assessed activities, which lead to potentially underestimate the overall patient’s functional level. In this case, the total score could lack of precision because it does not capture the real abilities of the patient. This issue is particularly noticeable when comparing IADL scores between men and women, considering that some tasks are more representative of men or women activities, as previously mentioned [11, 12]. Consequently, previous studies have proposed to differentiate the calculation of the score between men and women, or to calculate the score by using only some items (3, 4 or 5 depending on the study) of the IADL questionnaire less dependent of gender or more related to the risk of dementia or cognitive disorders, nevertheless, the reliability of such new scales has not been formally tested, and the choice of item varies between studies making comparison difficult [8, 9, 12–14]. Conversely, others publications have recommended to rate all items without differentiation for both genders [15, 16]. Given the different situations, it still appears complicate to decide how the Lawton IADL should be used in clinical practice as well as in research studies.

In this study, we propose a corrected score for IADL on a 8-point scale which includes the possibility that an activity was never carried out by an individual. The objectives of this article were to assess the relevance of the proposed corrected score and to measure the degree of agreement between the usual IADL score and its corrected version.

## Methods

### Study population and setting

The study population was selected from the observational multicenter MEMORA cohort including successive outpatients visiting memory centers of the Clinical and Research Memory Centre of Lyon (CMRR) (Trial registration: NCT02302482, registered 27 November 2014) [17]. The study sample included patients having undergone a medical examination between 2014 and 2017. The inclusion criteria were a subjective cognitive complaint, either expressed by the patient or one of its relative, at all stage of cognitive impairment, with or without a diagnosis of Alzheimer’s Disease or Related Diseases (ADRD), and having an evaluation of the IADL score. Non-inclusion criteria were an opposition to participate to the study, nursing home living, being under legal protection, or health conditions that do not allow carrying out clinical examinations based on questionnaires.

In terms of ethical and legal considerations, information has been individually provided to the patients and their informal caregivers at inclusion. They can oppose their participation in the research. The authorization for handling personal data has been granted by the national data protection commission.

### Usual and corrected IADL score

Functional abilities level was assessed using the Lawton IADL scale, which assesses 8 instrumental activities through 8 questions: ability to make telephone calls, to go grocery shopping, to prepare meals, to do housekeeping, to do laundry, to use transportation, to take medications, and to handle finances [8]. The IADL score corresponds to the sum of the binary answers at each question and varies from 0 (dependence) to 8 (autonomy). An additional answer “has never done” was added for each item of the IADL questionnaire to account for situation where a patient is not concerned by an activity.

A corrected IADL (C-IADL) was calculated in order to take into account the cases of patients who had never carried out one or more assessed abilities. C-IADL was given by the following formula:

$$ C- IADL=\left(\frac{IADL\ score}{8-a}\right)\ast 8 $$

where *a* corresponds to the number of activities never carried out in its life. Note that in the context of outpatient, it has been considered that everybody has used transport previously in its life. Consequently, *a* was only calculated on the 7 remaining activities, so that the denominator 8 − *a* could never be null. If a patient has never been concerned with laundry for instance, but presented autonomy on the 7 remaining activities, this patient had an usual IADL score equal to 7 on a 8-point scale and a C-IADL equal to 8 on a 8-point scale. This choice does not preclude measuring disability in this item since there is a possibility to answer “does not travel at all” or “Travel limited to taxi or automobile with assistance of another”.

### Other covariates

The collection of sociodemographic characteristics included age, gender, marital status (single, married/in couple, divorced/separated, widowed, unspecified), educational level (nil, primary, secondary, tertiary) and lifestyle (at home with spouse, at home with relatives, at home alone with relatives in the neighborhood, at home alone without relatives in the neighborhood, other lifestyle). Global cognition was assessed using the Mini Mental State Examination (MMSE), a brief cognitive screening instrument [18]. The MMSE yields to a single cognitive function score ranging from 0 (severe cognitive impairment) to 30 (no impairment). The diagnosis stage and etiologies were established by specialists (neurologist, geriatrician, or psychiatrist). The diagnosis stage of mild or major neurocognitive disorders (NCD) was identified using the DSM-V nomenclature [1]. The etiologies were identified as follows: Alzheimer’s disease (AD), AD with cerebrovascular component, cognitive disorders of vascular origin, Lewy body disease, frontotemporal degeneration, Parkinson’s disease and Parkinson’s syndroma, psychiatric disorders, other disorders, and no trouble.

The neuropsychiatric symptoms were assessed using the Neuropsychiatric Inventory (NPI) [19]. The NPI assesses 12 psychiatric or behavioral disturbances i.e. delusions, hallucinations, depression, agitation, anxiety, apathy, disinhibition, irritability, euphoria, aberrant motor behaviors, sleep disorders, and eating disorders. The total score ranges from 1 to 144 (more severe symptoms).

### Statistical analysis

Missing data were handled using the MICE method (Multivariate Imputations by Chained Equations) for marital status (5.6% of missing values), education (14.9%), lifestyle (0.1%), MMSE score (8.8%), and NPI score (17.8%) [20]. The IADL and C-IADL scores and all the above cited covariates were introduced in the imputation model.

Population characteristics were reported using the mean value and 95% CI for numeric variables, or counts and frequency for categorical variables. Mean values of usual IADL and C-IADL were described for each level of categorical covariates. The relationships between means of IADL or C-IADL and each variable were assessed using one-way ANOVA. The proportion of patients never concerned by one or several item of IADL was also reported overall and according to gender.

The Bland–Altman method was used to measure agreement between the usual IADL and C-IADL [21]. The first step of this method was to plot the difference between the two scores against their mean. The second step consisted in estimating the mean difference $\overline{d}$ and the standard deviation of the differences *s* in order to calculate the 95% limits of agreement, $\overline{d}-1.96\ s$ and $\overline{d}+1.96\ s$. If the differences are normally distributed, 95% of differences will lie between these limits. The agreement rate, i.e. the proportion of differences between the 95% limits of agreement was also calculated. This analyze was carried out in the overall sample and was also stratified by gender and diagnosis stage. The intra-class correlation coefficient (ICC) (“two-way mixed effects, absolute agreement, multiple raters/measurement”) was computed for the whole sample, by gender and diagnosis stage. The ICC was interpreted as poor agreement if ICC < 0.5: poor, moderate agreement if ICC is between 0.5 and 0.75, good agreement if ICC is between 75 and 0.9, and excellent agreement if ICC ≥0.9 [22, 23]. A multivariate logistic regression was performed to assess whether the characteristics of the patients i.e. age, gender, marital status, education, lifestyle, MMSE score, diagnosis stage, etiology stage, and NPI, may impact the agreement between the 2 scales. The outcome was a binary variable equal to 1 if the difference between the two scores lied between the 95% limits of agreement, 0 otherwise. A backward stepwise method was used to select significant variables. Adjusted Odds-ratios (OR), 95% CI and *p* value were reported.

Finally, multivariate linear regressions were performed to determine whether the same covariates explained the IADL and C-IADL scores, with similar effects. Two models were performed using alternatively the usual IADL and the C-IADL as dependent variable. Regression coefficients (b), standard error (SE) and *p*-value were reported.

A *p*-value < 0.05 was considered as statistically significant and all tests were bilateral. Analyses were performed using R software (version 3.5.0; R Foundation for Statistical Computing, Vienna, Austria).

## Results

### Characteristics of the study sample

The study population included 2391 patients, with a mean age of 79.9 (95% CI [79.6–80.2]) (Table 1). There was a majority of women (61.7%), 54.2% of the patients were married or in couple, while 31% were widowed; 53% of the patients lived at home with spouse, while 28.6% lived at home alone with their relatives in the neighborhood. Regarding medical conditions, the mean MMSE was 20.2 (95%CI = [19.2–20.4]) and 41.1% of the patients had a MMSE score inferior to 20. Patients with major neurocognitive disorders represented 40.8% of the population study and 42.2% of patients were concerned with ADRD. The mean NPI score was 17.4 (95%CI = [16.7–18.0]).

**Table 1:** Description of the usual IADL score and the C-IADL score according to the characteristics of the study population (*n* = 2391)

	n or mean	% or [95%CI]	Usual IADL	*p* value	C-IADL	*p* value
**Total**			4.25		4.61	
**Age (mean, 95% CI)**	79.89	[79.58–80.20]		< 0.001		< 0.001
**Gender**				< 0.001		< 0.001
Men	917	38.35	3.49		4.26	
Women	1474	61.65	4.72		4.82	
**Marital status**				< 0.001		< 0.001
Single	99	4.14	5.39		5.47	
Married/in couple	1296	54.2	3.87		4.45	
Divorced/separated	205	8.57	5.17		5.26	
Widowed	742	31.03	4.49		4.56	
Unspecified	49	2.05	4.53		4.86	
**Educational level**				< 0.001		< 0.001
Nil	443	18.53	3.47		3.73	
Primary	836	34.96	4.11		4.41	
Secondary	787	32.92	4.61		4.98	
Tertiary	325	13.59	4.81		5.40	
**Lifestyle at baseline**				< 0.001		< 0.001
At home with its husband/spouse	1266	52.95	3.92		4.53	
At home with relatives	160	6.69	3.16		3.24	
At home, alone, with relatives in the neighborhood	684	28.61	4.71		4.79	
At home, alone, without relatives in the neighborhood	200	8.36	5.63		5.68	
Other lifestyle	81	3.39	4.32		4.36	
**MMSE score (mean, 95% CI)**	20.18	[19.92–20.43]		< 0.001		< 0.001
[0,10[	157	6.57	1.92		2.12	
[10,20[	826	34.55	3.20		3.49	
[20,26[	828	34.63	4.54		4.92	
[26,30]	580	24.26	5.97		6.42	
**NPI score (mean, 95% CI)**	17.4	[16.76–18.04]		< 0.001		< 0.001
**Diagnosis stage**				< 0.001		< 0.001
Absence of trouble	16	0.67	5.13		5.90	
Subjective cognitive complaint	691	28.9	5.45		5.88	
Mild neurocognitive disorders	700	29.28	4.98		5.37	
Major neurocognitive disorders	975	40.78	2.86		3.13	
Other cases	9	0.38	5.00		5.25	
**Etiology**				< 0.001		< 0.001
Etiology not yet established	1107	46.3	4.82		5.25	
Alzheimer’s disease	576	24.09	3.28		3.51	
Alzheimer’s disease with cerebrovascular component	131	5.48	2.99		3.31	
Cognitive disorders of vascular origin	198	8.28	3.38		3.75	
Lewy body disease	41	1.71	2.95		3.36	
Fronto temporal dementia	10	0.42	1.50		1.65	
Parkinson’s disease, and Parkinson’s syndroma	54	2.26	3.22		3.65	
Psychiatric disorders	132	5.52	5.81		6.11	
Other disorders	78	3.26	4.46		4.94	
No trouble	64	2.68	7.02		7.26	

*C-IADL* corrected Instrumental Activities of Daily Living score, *IADL* Instrumental Activities of Daily Living score, *MMSE* Mini-Mental Stage Examination, *NPI* Neuropsychiatric Inventory, *ref.* reference category

*p* value from a one-way Anova

### Distribution of IADL and C-IADL according other covariates

Overall, the mean IADL and the mean C-IADL were 4.25 and 4.61, respectively. Women had a mean IADL higher than men: 4.72 vs 3.49. This difference between women and men decreased when considering the mean C-IADL: 4.82 vs 4.26. The mean IADL was also higher for single or widowed compared to married/in couple patients: 5.39 and 5.17 respectively, vs 3.87. This difference between single, widowed and married patients decreased when considering the mean C-IADL: 5.47, 5.26, and 4.45, respectively. The usual IADL and the C-IADL scores increased with the educational level. The functional autonomy increased with the MMSE score, and the two mean scores IADL and C-IADL were lower for patients with major cognitive disorders and/or with ADRD.

### Gender disparities on instrumental daily living activities

On the overall study population, 36.9% of patients had never carried out at least one activity (Table 2). This proportion was different according to gender: more men had never carried out at least one activity (68.8%) compared to women (17%). Regarding the detailed item of IADL, the higher differences between men and women were for the item “has never done their laundry”, with a large difference between men (61.4%) and women (0.6%), “has never prepared meals” (33.1% vs. 0.3% respectively), and “has never done housekeeping” (25.9% vs. 0.2% respectively). Disparities were less pronounced for the other activities.

**Table 2:** Description of answers in IADL item according to gender

	Total		Men		Women	
	*n* = 2391	%	*n* = 917	%	*n* = 1474	%
**Number of answers “has never carried out … “**						
0	1509	63.11	286	31.19	1223	82.97
At least 1 activity	882	36.88	631	68.81	251	17.03
**Detailed answers by item of IADL**^a^						
Has never used phone	10	0.42	7	0.76	3	0.2
Has never taken his/her medicine	48	2.01	24	2.62	24	1.63
Has never handle his/her finance	400	16.73	178	19.41	222	15.06
Has never done laundry	572	23.92	563	61.4	9	0.61
Has never done shopping	70	2.93	66	7.2	4	0.27
Has never prepared meals	309	12.92	304	33.15	5	0.34
Has never done housekeeping	240	10.04	237	25.85	3	0.2

^a^ note that the item transportation is not included as everyobody has used it once

### Agreement measurement between the two scores

**Fig. 1:**
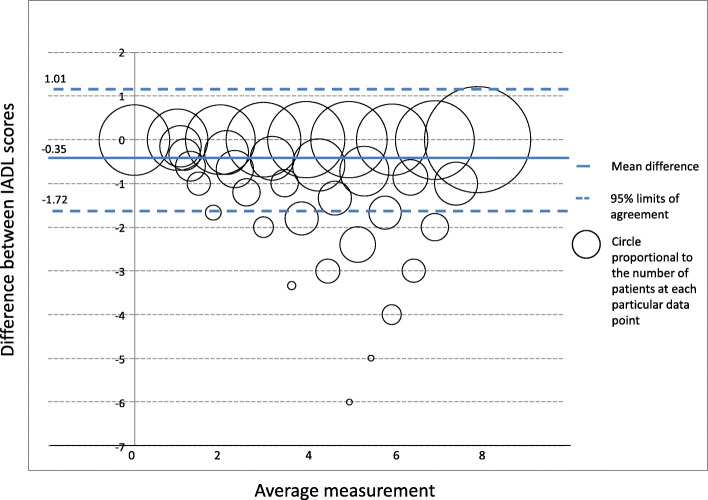
Bland–Altman plot for the overall study population

With the Bland-Altman analysis, the mean difference between the two scores (IADL and C-IADL) was − 0.35 (SD = 0.7) (Table 3). Overall, 93.5% of observations lied within the 95% limits of agreement [− 1.72–1.01]. The observations which were outside the limits interval had a mean ranging from 3 to 7 (Fig. 1). The difference between the two scores tended to get larger as the mean increased. In the stratified analysis by gender, the mean difference was larger in women (− 0.77, SD = 0.9) than women (− 0.1, SD = 0.3). Almost 95% of the observations for male lied within the 95% limits of agreement compared to 93% of the observations for women, nevertheless the limits of agreement for men were larger than those for women. In the stratified analysis by diagnosis stages, the difference between the two methods also increased with the mean increase, and proportion from 93 to 100% of observations lied within the 95% limits of agreement depending of the diagnosis stages.

**Table 3:** Indicators derived from Bland-Altman analyses

	Mean difference (d)	SD	Lower limits agreement	Upper limits agreement	% of observations inside limits agreement	ICC	95% CI ICC
**Total**	−0.35	0.70	−1.72	1.01	93.52	0.98	0.978–0.982
**Sex**							
Men	−0.77	0.93	−2.61	1.07	94.77	0.96	0.956–0.966
Women	−0.10	0.25	−0.59	0.40	93.28	0.99	0.997–0.998
**Diagnosis**							
Absence of trouble	−0.77	0.97	−2.68	1.13	93.75	0.93	0.801–0.976
Subjective cognitive complaint	−0.42	0.79	−1.97	1.13	92.90	0.97	0.962–0.972
Mild Neurocognitive disorder	−0.40	0.76	−1.97	1.12	93.71	0.97	0.966–0.975
Major neurocognitive disorder	−0.27	0.55	−1.35	0.81	94.46	0.98	0.98–0.984
Other case	−0.25	0.43	−1.10	0.60	100.00	0.99	0.965–0.998

*SD* standard deviation of the mean difference, *ICC* intra-class correlation coefficient

The ICC analysis showed a coefficient at 0.98 indicating an excellent agreement between the 2 scores. In the stratified analyses by gender and diagnosis staged, the ICC ranged from 0.93 to 0.99 depending on the groups.

### Sensitivity of agreement to patients’ characteristics

In the multivariate logistic regression model, female gender, living at home with spouse or at home alone with their relatives in the neighborhood, as well as major neurocognitive disorders were mutually associated with increased chance to be within the limits agreement (Table 4). The variables of age, marital status, educational level, MMSE, NPI and etiology did not contribute significantly and were excluded of the model.

**Table 4:** Adjusted odds-ratio for agreement modelisation

	Adjusted OR^b^	95% CI		*p* value
**Gender**				
Men	ref.			
Women	86.024	21.121	350.367	< 0.000
**Lifestyle at baseline**				
At home with its husband/spouse	ref.			
At home with relatives	10.173	1.347	76.834	0.025
At home, alone, with relatives in the neighborhood (and other lifestyle)^a^	16.947	4.13	69.54	< 0.000
At home, alone, without relatives in the neighborhood	4.074	0.951	17.458	0.058
**Diagnosis stage**				
Absence of trouble	0.338	0.081	1.403	0.135
Subjective cognitive complaint	ref.			
Mild neurocognitive disorders (and other cases)^a^	1.127	0.737	1.722	0.581
Major neurocognitive disorders	2.535	1.597	4.024	< 0.000

*ref.* reference category

^a^ 2 categories with similar mean IADL were regrouped to manage small sizes

^b^ Variables excluded from the model: age, marital status, educational level, MMSE, NPI, etiology

### Relationships between patients’ characteristics and functional level assessed with both scores

Results of the two multivariate linear regressions showed similar relationships between patients’ characteristics and the functional level measured either with the usual IADL or C-IADL, excepted for the gender i.e. the estimated effect associated to women was lower in comparison to male when modelling C-IADL (b = 0.55, SE = 0.02) than the estimated effect when modelling the usual IADL score (b = 1.09 SE = 0.09) (Table 5).

**Table 5:** IADL and C-IADL modelisation (linear regression)

	Outcome = IADL			Outcome = C-IADL		
	b^a^	SE	*P* Value	b^a^	SE	*P* value
**Age**	−0.054	0.005	< 0.0001	−0.054	0.006	< 0.0001
**Gender**						
Men	ref.			ref.		
Women	1.089	0.085	< 0.0001	0.545	0.09	< 0.0001
**Marital status**						
Married/in couple	ref.			ref.		
Single	0.755	0.235	0.001	0.618	0.247	0.012
Divorced/separated	0.671	0.188	< 0.0001	0.53	0.197	0.007
Widowed	0.635	0.16	< 0.0001	0.599	0.168	< 0.0001
Unspecified	0.506	0.267	0.058	0.349	0.28	0.214
**Educational level**						
Nil	ref.			ref.		
Primary	0.187	0.109	0.085	0.202	0.114	0.077
Secondary	0.318	0.112	0.005	0.324	0.118	0.006
Tertiary	0.313	0.142	0.027	0.333	0.149	0.025
**Lifestyle at baseline**						
At home with its husband/spouse	ref.			ref.		
At home with relatives	−0.935	0.196	< 0.0001	−1.186	0.206	< 0.0001
At home, alone, with relatives in the neighborhood and other lifestyle	−0.032	0.161	0.844	−0.291	0.169	0.085
At home, alone, without relatives in the neighborhood	0.423	0.197	0.031	0.162	0.206	0.432
**MMSE**						
[0,10[	−2.282	0.186	< 0.0001	−2.421	0.195	< 0.0001
[10,20[	−1.535	0.119	< 0.0001	− 1.56	0.125	< 0.0001
[20,26[	−0.865	0.104	< 0.0001	− 0.863	0.11	< 0.0001
[26, 30]	ref.			ref.		
**NPI score**	−0.03	0.002	< 0.0001	−0.03	0.003	< 0.0001
**Diagnosis stage**						
Subjective cognitive disorders	ref.			ref.		
Mild neurocognitive disorders and other cases	0.056	0.103	0.589	0.032	0.108	0.768
Major neurocognitive disorders	−0.879	0.122	< 0.0001	− 0.982	0.128	< 0.0001
Absence of trouble	0.167	0.462	0.718	0.411	0.486	0.397
**Etiology**						
Alzheimer’s disease	ref.			ref.		
Alzheimer’s disease with cerebrovascular component	−0.188	0.174	0.283	− 0.124	0.183	0.498
Cognitive disorders of vascular origin	−0.343	0.151	0.023	−0.358	0.158	0.024
Lewy body disease	−0.006	0.291	0.983	0.103	0.305	0.736
Fronto temporal dementia	−1.904	0.579	0.001	−2.165	0.608	< 0.0001
Parkinson’s disease, and Parkinson’s syndroma	− 1.034	0.26	< 0.0001	− 1.084	0.273	< 0.0001
Psychiatric disorders	0.416	0.189	0.028	0.433	0.198	0.029
Other disorders	−0.033	0.224	0.883	0.054	0.236	0.817
Etiology not yet established	0.412	0.109	< 0.0001	0.498	0.115	< 0.0001
No trouble	1.238	0.259	< 0.0001	1.127	0.272	< 0.0001

*C-IADL* corrected Instrumental Activities of Daily Living score, *IADL* Instrumental Activities of Daily Living score, *MMSE* Mini-Mental Stage Examination, *NPI* Neuropsychiatric Inventory, *ref.* reference category

^a^ b: regression coefficient

## Discussion

In this large study population, we found that the corrected IADL score had an excellent degree of agreement with the usual IADL score based on an ICC equal to 0.98, while Bland and Altman method showed that 93.5% of observations lied within the 95% limits of agreement. The level of agreement between the usual IADL and the corrected IADL varies little according to gender and diagnosis stage. This study also showed that 36.9% of patients had never carried out at least one of the seven abilities evaluated in the IADL questionnaire (transportation being always at least carried out once), and large disparities were observable between gender, justifying the need to adjust the IADL score calculation to ensure its clinical relevance. Analyses by item of the IADL scores also highlighted that, in this study population, men were less frequently concerned by some abilities such as doing laundry, preparing meals and doing housekeeping compared to men. The proposed correction of the IADL score showed a reduction to the difference of the global score between men and women and provides a more accurate reflection of reality: a patient who has never carried out an ability being count has if he/she did not have this ability in the usual IADL score, which leads to a possible underestimation of the level of overall functional autonomy. Another interesting finding is that the same factors were related to the two IADL scores, the direction and the magnitude of the estimated effects being quite similar.

### Strengths and weaknesses of the study

To our knowledge, this study is the first to provide a simple and intuitive correction of the usual IADL that ensures the real clinical state of the patient. This correction scored is easy to calculate in clinical setting and for research purpose and would simply imply the adding of the answer “has never done” at each item of the IADL questionnaire. As the corrected IADL score is scored on 8 points for all the patients, as the usual score, this facilitates its interpretation from a patient to another for both physicians and research: patients with an identical IADL score might not be at the same functional level depending on whether this score was calculated with the answer to the 8 questions or less. Another strength of the study is the evaluation of the corrected score in the context of current practice in a large population of patients at all stages of NCD or with subjective cognitive complaint which allows to expand the scope of these results. This analysis was able to take into account a wide range of patients’ characteristics which could be related with the degree of agreement between the two scores.

In this study, the proportion of observations within the limits agreement was slightly inferior to 95% which is the threshold that was recommended by Bland and Altman to consider that two measures are commutable. The Bland and Altman plot also shows a negative bias. The question that may arise is whether this correction could not overestimate the functional abilities if we consider that patients who have never done some of the activities could have lower functional level compared to those who have already done them. There is no evidence that a patient who has never done an activity would be able to do it if he/she had to do it. Thus, we hypothesize that the fact to have never done some of the activities could be related to individual preferences or living habits (a person living in couple with another person who accomplishes the task). This hypothesis is supported by additional analyses of our data. For example, proportions of men who had never done these activities were higher when they lived with someone compared to when they lived alone: 40.3% of men living with someone had never prepared meals vs. 6.8% in men living alone, respectively 30.3% vs. 8.3% for having never done housekeeping. Furthermore, the comparison of the cognitive performance (MMSE) between patients who had never prepared meals or who had never done housekeeping with patients who had already done these activities showed that both groups had similar cognitive performance (respective *p* values for difference *p* = 0.2 and *p* = 0.5) (detailed results now shown). In contrast, patients who were unable to perform these tasks were much more cognitively impaired than those who had never done it (*p* values for difference *p* <  0.0001 for meals preparation and housekeeping). Thus, one possible explanation of the negative bias observed in the Bland and Altman plot is that the estimation of the functional abilities level with the usual IADL is precisely affected by a systematic bias measurement due to gender difference. The corrected IADL could then allow correcting this bias.

The present study was conducted in a population of older adults attending a memory center; their characteristics are different from the general population with higher proportion of patients with neurocognitive disorders and functional limitations, studies conducted in others populations would therefore be needed to assess the relevance of the proposed corrected score in different contexts.

### Comparison with the literature

Our findings are consistent with previous studies which have observed item response bias and in particular gender bias in the Lawton IADL scale, the overall score could reflect in some cases more a living context than real functional limitations [8, 11, 24–26]. Besides, in Hesseberg et al. the difference between gender was only found for the item “laundry” [27]. Though, there is still a lack of a standard way of measurement of the IADL, and while some tasks appear more specific for women, they can also concern men and excluding some of them in routine care could constitute an a priori subjective judgment that could interfere with the results. Our findings highlighting similar relationships between the patients’ characteristics and the usual IADL score, and the corrected IADL score confirm and extend the results of previous studies conducted in community-dwelling study population, or which used only a limited selection of items included in the IADL questionnaire [14, 28, 29]. In terms of patient’s characteristics, our study sample differed slightly to the larger sample of the French National Alzheimer (BNA) database registers in memory centers in France (mean age 79.9 years old vs. 77.4 to 81.3 in the BNA depending on the selection; 62% of women vs. 63.2 to 70.6% in the BNA; 29.6% of patients with probable AD vs. 27.3 to 29.9% in the BNA, mean MMSE 20.2 vs. 16.8 to 25.7 in the BNA) [28, 30].

## Conclusions

This study proposes a simple correction of the usual IADL score which has an excellent reliability, and allows to take into account the possibility that patients have never done one or several assessed activities. As the Lawton IADL is often used to evaluate the diagnosis stage of the neurocognitive disorders of patients in memory centers, this corrected version could benefit both for the clinical practice by providing a more accurate description of the real clinical state of the patients allowing to manage them more precisely, and for research involving the evaluation of the functional abilities of patients such as those assessing the efficacy of interventions on the limitation of functional loss. Additional researches should be performed to confirm these results in others populations and to assess the sensibility to change of the corrected score.

## Data Availability

The study dataset cannot be publicly available due to regulations and agreements obtained to perform the MEMORA study. Data requests can be submitted to the researchers at the Memory Research Centre of Lyon (CMRR of Lyon, Charpennes Hospital, University Hospital of Lyon, Villeurbanne, France).

## Abbreviations

AD: Alzheimer’s disease
ADRD: Alzheimer’s Disease or Related Diseases
DSM-V: Diagnosis and Statistical Manual of mental disorders criteria
ICC: Intraclass correlation coefficient
IADL: instrumental activity of daily living
MICE: Multivariate Imputations by Chained Equations
MMSE: Mini-Mental State Examination
NCD: Neurocognitive disorders
